# Editorial: Occupational health psychology: From burnout to well-being at work

**DOI:** 10.3389/fpsyg.2022.1069318

**Published:** 2022-11-14

**Authors:** Sónia P. Gonçalves, Joana Vieira dos Santos, Hugo Figueiredo-Ferraz, Pedro R. Gil-Monte, Mary Sandra Carlotto

**Affiliations:** ^1^Centro de Administração e Políticas Públicas, Instituto Superior de Ciências Sociais e Políticas, Universidade de Lisboa, Lisbon, Portugal; ^2^Instituto Superior de Ciências Sociais e Políticas, Universidade de Lisboa, Lisbon, Portugal; ^3^University of Algarve, Faro, Portugal; ^4^Valencian International University, Valencia, Spain; ^5^University of Valencia, Valencia, Spain; ^6^Conselho Nacional de Desenvolvimento Científico e Tecnológico (CNPq), Brasília, Brazil; ^7^Associação Nacional de Pesquisa e Pós-graduação em Psicologia, Brasília, Brazil

**Keywords:** occupational health psychology, burnout, workplace, wellbeing, individual characteristics, organizational characteristics

This Research Topic entitled *Occupational Health Psychology (OHP): From Burnout to Well-being at Work* tried to bring together two applied disciplines within psychology: health psychology and industrial/organizational psychology. OHP applies the psychology principles to protect health, improve the quality of work life and promote the health and wellbeing of workers (Gil-Monte, 2014). The notion of health protection in this definition refers to the analysis and interventions to reduce the toxicity of workplaces and diminish the employees demands that could impact on their health (e.g., burnout). Moreover, health promotion refers to analyses and interventions that focus on providing individuals with diverse resources to deal with different types of environments so as to guarantee their wellbeing, while at the same time encouraging healthy work environments. The workplace is a privileged context of action to promote health (Commission of the European Communities, 2005). The intense transformation of work and employment in the past decades has been contributing to changes in organizational structures and processes that are influencing the health and wellbeing of workers in their work roles, as well as in their personal and families' roles and boundaries (International Labour Organization, 2016).

The challenge of OHP in the future is to explore the positive mechanisms and simultaneously continue to study the illness and distress felt by this cohort. Both approaches are complementary and only jointly can allow a more integrative and complete understanding of the relationships between work, organization, and workers. It was an honor for us to promote such an interesting Research Topic. The number of articles (21 articles published) and the dissemination of the thematic, which includes 93 authors from more than ten countries, is the scientific validation of interest in the theoretical and empirical rationale of the current Research Topic.

The simple analysis of key-words and samples allows for the understanding of the diversity and richness of this Research Topic (Table 1), providing important contributions toward the field of occupational health psychology.

**Table 1:** Articles key-words and samples.

**Authors**	**Key-words**	**Sample**
Yin et al.	Total rewards perceptions, kindergarten teachers, organizational identification, work engagement, mediating effect	1,014 kindergarten teachers
Zhang X. et al.	Burnout, COVID-19, healthcare workers, MBI scale, frontline healthcare workers	1,163 healthcare workers
Martínez et al.	Personality, affect, coping strategies, nurses, descriptive of survey study	1,268 nurses
Baka and Prusik	Hindrance and challenge stressors, job burnout, work-family conflict, nurses, mediation effect	516 nurses
Tolonen et al.	Compassion, personality, job demand control, effort-reward imbalance, longitudinal	723 workers ≠ occupations
Makara-Studzinska et al.	Emergency service, self-efficacy, occupational burnout, perceived stress, COVID-19, emergency call-taker and dispatcher	546 call-takers and dispatchers
Zhang F. et al.	Organizational identification, psychological capital, job satisfaction, income level, residents	310 residents
Mishima-Santos et al.	Work design, wellbeing, remote work, teleworker, work from home, work characteristics, multimethod, COVID-19	1,108 teleworkers
Damasceno et al.	Work teams, innovation, emotion, emotional carrying capacity, affective commitment	138 Portuguese work teams ≠ sectors of activity
Cook and Zill	Diabetes mellitus, burnout, job satisfaction, distress, chronic illness	297 participants ≠ occupations
Gulla and Golonka	Sensory processing sensitivity, highly sensitive person, highly sensitive person scale, attention awareness, mindfulness, resilience	273 participants ≠ occupations
Tikkanen et al.	Burnout, drop-out, PhD candidate, research engagement, wellbeing	692 PhD candidates in medicine
Golonka and Gulla	Burnout, sensory processing sensitivity (SPS), highly sensitive person, Oldenburg Burnout Inventory (OLBI), Highly Sensitive Person Scale (HSPS), wellbeing	516 employees ≠ occupations
Xie et al.	Job demands, job resources, burnout, psychological distress, social workers, China	897 social workers
Upadyaya et al.	Stress, latent profile analysis (LPA), school principals, COVID-19, demands and resources	535 school principals
Figueiredo-Ferraz et al.	Burnout, feelings of guilt, psychosomatic disorders, occupational stress, teachers	1,266 teachers
Matsushita and Yamamura	Occupational health, mental health, stress response, overtime work, school teachers, job title	54,772 teachers
Li and Wu	Education, underemployment, health, endogeneity, labor	10,563 participants ≠ occupations
Zhang S. et al.	Medical students, learning passion, self-esteem, psychological capital, professional identity	1,218 medical students
Makowska-Tłomak et al.	Digital transformation stress, digital transformation, online intervention, self-efficacy, burnout, COVID-19	558 participants ≠ occupations
Yin et al.	Burnout, time-to-treatment, social support, type D personality, decision making	1,039 general participants

The first contribution by Ji and Cui explores the observed relations between rewards perceptions and work engagement of kindergarten teachers, and the mediating role of organizational identification. The main results show that organizational identification partially mediated the relationship between total rewards perception and work engagement.

The second contribution, published by Zhang X. et al., shows that burnout is prevalent among frontline healthcare workers fighting COVID-19. This study also explores the factors associated with burnout.

From Spain, the authors Martínez et al.bring a study that aims to analyze the relationships between the personality, positive and negative affects, and the coping strategies of nurses. Negative affects were a mediator in the relationship between personality and less adaptative strategies, whilst positive affects had a strong impact on the development of positive strategies.

The 1-year cross-lagged Baka and Prusik study investigates the effects of five types of job demands related to challenge and hindrance stressors on job burnout as well as the mediational role of work family conflict in Polish nurses.

The study *Rewards of compassion: Dispositional compassion predicts lower job strain and effort-reward imbalance over a 11-year follow-up* developed by Tolonen et al. examines (i) whether dispositional compassion predicts job strain and effort-reward imbalance (ERI) or the predictive relationship run from job strain and ERI to dispositional compassion and (ii) the effect of dispositional compassion on the developmental trajectory of job strain and ERI over a 11-year follow-up (2001–2012).

The study by Makara-Studzinska et al. is focused on the relationship between stress, burnout and employee personal resources in a sample of 546 call-takers and dispatchers from 14 public-safety answering point in Poland.

The study published by Zhang F. et al. analyzed the mediating effect of organizational identification on the relationship between psychological capital and job satisfaction.

Mishima-Santos et al. presented a study that analyzed work characteristics in remote work to wellbeing, using a two-stage multi-method approach.

The aim of the research published by Damasceno et al. is centered on the relationship between emotional carrying capacity and group innovation, considering affective commitment as the mediating variable with a sample composed of 625 members and their respective leaders.

Cook and Zill investigated the association between diabetes-related distress and work outcomes (burnout and job satisfaction) among employed people with type 1 diabetes.

The study published by Gulla and Golonka shows significant relationships between sensory processing sensitivity and resilience.

Tikkanen et al.'s study focused on exploring individual variations in medicine doctoral candidates' wellbeing, in terms of experienced research engagement and burnout by using a person-centered approach.

A study with social workers developed by Xie et al. supported a dual process by which JD-R affected both social workers' burnout and psychological distress through health impairment and motivation processes.

Upadyaya et al. study examined three latent profiles of school principals' stress concerning stress concerning the ability of students, teachers, parents and principals to cope during the COVID-19 pandemic: *high stress, altered stress* and *low stress*.

The results obtained in the study by Figueiredo-Ferraz et al. provide empirical evidence for the mediator role of guilt in the relationship between the burnout syndrome and psychosomatic disorders in a sample of teachers from Spain and Portugal, and they contribute to the empirical validation of the model of burnout developed by Gil-Monte (Gil-Monte, 2005).

The contribution of Matsushita and Yamamura examines the working hours of junior high school teachers in public schools and investigates the association between overtime work and stress responses across job titles.

Results from Li and Wu show that underemployment is significantly related to the decline of self-rated health, increased depressive tendencies, and the prevalence of illness over a certain period.

The Cross-Sectional Study of Individual Learning Passion in Medical Education developed by Zhang S. et al. demonstrates that self-esteem significantly and positively influenced medical students' PsyCap, which fully mediated the relationship between self-esteem and harmonious learning passion.

Makowska-Tłomak et al. bring a contribution related with a Blended Online Intervention based on cognitive behavioral therapy and social cognitive therapy.

Finally, Yin et al.'s study shows, for instance, that age, job burnout and worrying about expenditure were the three determinants for prehospital decision delay.

The Research Topic “*Occupational health psychology: From burnout to well-being at work*” presents considerable wealth in terms of the heterogeneity of authors and countries with contributions. In addition, the diversity of professional groups covered in the research and thematic areas is significant. We consider, therefore, that we have successfully achieved our objective by providing an opportunity for the sharing of interesting results and future avenues of research and applied implications regarding Occupational Health Psychology. The new avenues of research in this area need the contribution of each one of you for their recognition, not least because the improvement of work environments contributes to the health and wellbeing of workers and the healthy performance of organizations.

## Author contributions

All authors listed have made a substantial, direct, and intellectual contribution to the work and approved it for publication.

## Conflict of interest

The authors declare that the research was conducted in the absence of any commercial or financial relationships that could be construed as a potential conflict of interest.

## Publisher's note

All claims expressed in this article are solely those of the authors and do not necessarily represent those of their affiliated organizations, or those of the publisher, the editors and the reviewers. Any product that may be evaluated in this article, or claim that may be made by its manufacturer, is not guaranteed or endorsed by the publisher.
